# Traditional Chinese Medical Care Application Patterns Among Nurses With Menstrual Symptoms: A Q Methodology Investigation

**DOI:** 10.1002/nop2.70644

**Published:** 2026-07-15

**Authors:** Shu‐He Huang, Shu‐Chuan Yu, Shun‐Fen Chen, Yin‐Chen Chen, Hsiao‐Pei Hsu, Chiu‐Mieh Huang

**Affiliations:** ^1^ Department of Nursing, College of Nursing National Yang Ming Chiao Tung University Taipei Taiwan; ^2^ Department of Nursing Cardinal Tien Hospital New Taipei City Taiwan; ^3^ Department of Nursing Taipei Veterans General Hospital Taipei Taiwan; ^4^ Institute of Clinical Nursing, College of Nursing National Yang Ming Chiao Tung University Taipei Taiwan

**Keywords:** menstrual symptoms, nurses, Q methodology, traditional Chinese medicine

## Abstract

**Aim:**

To identify patterns of traditional Chinese medicine (TCM) care application among nurses experiencing menstrual symptoms.

**Design:**

This study employs Q methodology, where participants ranked statements through Q sorting based on their perceptions. The data were analysed using principal component analysis to identify shared perspectives on the use of TCM for managing menstrual symptoms among nurses.

**Methods:**

This study involved 60 nurses to explore their perspectives on the use of TCM care for managing menstrual symptoms. A set of 41 Q‐statements was formulated through a comprehensive literature review and expert assessment. Data were gathered via an electronic platform and analysed using PQMethod 2.35 software.

**Results:**

The final model identified five patterns, which accounted for 44.14% of the total variance. These patterns are as follows: (1) Expecting pain relief without painkillers; (2) prioritizing a healthy lifestyle; (3) trusting TCM for menstrual and reproductive health; (4) relying on TCM for pain management and daily activities; and (5) exploring various TCM treatments for beneficial outcomes.

**Conclusions:**

This study explored the perspectives of nurses who use traditional Chinese medicine (TCM) to manage menstrual symptoms. By focusing on nurses as both professional caregivers and individuals dealing with menstrual symptoms, this study identified five shared perspective patterns related to how nurses incorporate TCM care within the occupational context.

**Patient or Public Contribution:**

This study did not involve patient participation.

## 1 Introduction

During childbearing years, women frequently experience prevalent menstrual symptoms (Armour, Parry, Al‐Dabbas, et al. 2019). These symptoms, including menstrual pain, lower back pain, bloating, sore breasts, headaches, tiredness, mood swings and irritability, significantly impact physical comfort and quality of life (Bruinvels et al. 2021; Schoep et al. 2019). Severe menstrual symptoms can interfere with social functioning, learning and work efficiency, contributing to poorer mood and sleep quality (Bruinvels et al. 2021; Kho and Shields 2020; Ray et al. 2023). Nursing, as a highly demanding profession with shift work, places additional strain on women who already experience menstrual symptoms. Research shows that many nurses suffer from irregular menstrual cycles and abnormal endocrine function, leading to heightened symptoms such as pain and irregular menstruation (Cheng et al. 2019; Park et al. 2019; Yöndem and Çıtak Bilgin 2020). Given their pivotal role in healthcare and the unique challenges they face, nurses with menstrual symptoms represent a particularly vulnerable group that warrants focused attention and support.

Menstrual conditions such as premenstrual syndrome and dysmenorrhea are highly prevalent among women and can substantially impair both quality of life and work performance (Yang et al. 2025; Yoshino et al. 2022). Some nurses rely on over‐the‐counter pain relief medication to manage menstrual cramps, allowing them to remain productive at work (Armour, Parry, Al‐Dabbas, et al. 2019). Some nurses may incorporate traditional Chinese medicine (TCM) to address menstrual symptoms. Studies show that women frequently turn to traditional and complementary approaches—such as heat therapy, herbal remedies, massage and over‐the‐counter analgesics—to manage menstrual discomfort (Uçak and Süzer Özkan 2022). TCM is commonly utilized for the alleviation of menstrual symptoms. Although a growing body of research has examined TCM for menstrual symptoms, the existing literature has focused predominantly on the clinical efficacy, safety and underlying mechanisms of specific TCM interventions, including herbal formulas, acupuncture, heat therapy and other external therapies (Chen et al. 2024; Zhou et al. 2025). Systematic reviews and meta‐analyses have synthesized the comparative effectiveness of these approaches in relieving dysmenorrhea, premenstrual symptoms and related discomforts (Chen et al. 2024; Xie et al. 2026). Collectively, these studies have strengthened the evidence base for TCM as a therapeutic option for menstrual symptom management.

Women seek TCM to manage menstrual discomfort, with the expectation not only of alleviating pain and associated symptoms but also of enhancing fertility (Sosorburam et al. 2019; Tsai et al. 2016). The widespread use of TCM has become an integral component of menstrual health maintenance among women. However, limited attention has been given toward understanding how women perceive, interpret and incorporate TCM into their daily lives, particularly within occupational contexts characterized by high work demands. Nurses embody dual roles as healthcare providers and individuals managing their own menstrual symptoms, making their perspectives uniquely valuable for examining the integration of culturally embedded practices such as TCM. The World Health Organization recognizes menstrual health as a multidimensional issue encompassing physical, psychological and social well‐being, as well as a fundamental human rights concern (World Health Organization 2022). Previous research conducted in hospital settings indicates that nurses experiencing dysmenorrhea often continue to work despite pronounced discomfort (Jung et al. 2024). However, institutional support for menstrual health remains insufficient, leaving menstruating nurses to depend largely on personal coping strategies rather than organizational resources. This critical gap in understanding nurses' lived experiences of TCM use within the caregiving‐self‐care connection represents a practical oversight. Nurses' experiences uniquely illuminate the tension between professional healthcare responsibilities and personal health needs. Accordingly, this study addresses this gap by employing Q methodology to systematically explore nurses' subjective perspectives on TCM use for menstrual symptom management, emphasizing the interplay between their professional practice and personal well‐being. These insights hold potential to inform targeted nursing workplace policies and culturally sensitive menstrual health interventions.

### 1.1 Aim

To identify patterns of TCM care application among nurses experiencing menstrual symptoms.

## 2 Methods

### 2.1 Methodology Background

To explore nurses' subjective perspectives on the use of TCM for managing menstrual symptoms, this study employed Q methodology, a systematic approach for investigating human subjectivity in a structured and interpretable manner. Q methodology is particularly well suited to the study of complex viewpoints because it enables researchers to identify both shared patterns and meaningful differences in participants' attitudes, beliefs and preferences (Watts and Stenner 2012). Unlike conventional qualitative methods, such as interviews or thematic analysis, which typically generate rich narrative accounts but may be less effective in systematically comparing viewpoints across participants, Q methodology allows participants to rank a set of statements according to their subjective importance and then uses by‐person factor analysis to reveal clusters of individuals who share similar perspectives (Brown 1993). In contrast to conventional quantitative methods, such as surveys with predefined response categories and variable‐centered statistical analyses, Q methodology does not seek to generalize findings to a wider population or estimate the prevalence of particular attitudes. Instead, it is designed to identify distinct configurations of viewpoints within a group (Watts and Stenner 2012). Because it combines the qualitative expression of subjectivity with quantitative analytic procedures, Q methodology is often regarded as an integrative or mixed‐methods approach (Buchholtz and Vollstedt 2024; Ramlo 2020). This feature makes it particularly well‐suited to the present study, where nurses' decisions regarding TCM use for menstrual symptom management may reflect a complex combination of personal symptom experiences, healthcare responsibilities, workplace constraints and cultural beliefs. Q methodology has increasingly been applied in healthcare research to examine complex professional and patient perspectives, including in nursing education (Hensel et al. 2022), healthcare professionals' attitudes toward patients with fibromyalgia (Scott et al. 2023) and healthcare professionals' adoption of clinical technologies (Ladan et al. 2018), supporting its value for uncovering diverse yet patterned viewpoints in healthcare contexts.

We applied the Checklist for Reporting a Q Methodology Study developed by Dieteren et al. (2023). The checklist covers the four phases of a Q methodology study: study design, data collection, data analysis and presentation of findings (Table 1).

**TABLE 1:** Reporting checklist for the Q methodology study.

Topic		Descriptions	Page number
1.	Study design		
1.1	Source(s) of the concourse	The Q statements were adapted from the research team's prior study (Tsai et al. 2016)	9
1.2	Item sampling process	An initial pool of 134 statements was yielded from the qualitative data of previous work, which researchers refined by consolidating similar items and eliminating duplicates, resulting in 57 statements. Five experts then evaluated the content validity of the revised statements. Finally, 41 statements with content validity index (CVI) values > 0.8 were retained	10
1.3	Type of items	All 41 Q statements were textual statements without any visual items	10
1.4	Number of items	41 Q‐statements	10
1.5	Pilot study	No pilot study	Not applicable
2.	Data collection		
2.1	Selection of participants	Because prior use of TCM treatments for menstrual symptoms was a key inclusion criterion, other sampling methods may not be feasible; therefore, convenience and snowball sampling were employed	9
2.2	Number of participants	We recruited 60 eligible nurses through advertisements posted on hospital websites and through snowball sampling	9
2.3	Mode of interview	Upon completion of the Q‐sort, participants were invited to provide brief rationales for their selections in the extreme categories (+3 to +4 and −3 to −4)	12
2.4	Type of interview	Face‐to‐face	12
2.5	Ranking instruction	Participants were first guided to review the list of 41 Q statements and mark those they agreed with (+), disagreed with (−), or felt neutral toward (no mark). The research team then provided participants with laptop computers to perform the Q‐sort on the e‐platform	11–12
2.6	Sorting grid	The Q‐sort grid used in this study is shown in Figure 1	Figure 1
3.	Data analysis		
3.1	Factor analysis	Factor extraction and rotation were conducted using principal component analysis with varimax rotation	12
3.2	Factor selection	We adopted a five‐factor solution, as all eigenvalues were above 1 and the scree plot slope began to level off after the fifth factor	13
3.3	Bipolar factors	An absolute factor loading of one Q‐sort exceeded the 0.40 threshold, but the loading itself was negative and was therefore removed manually	13
3.4	Interpretation of the factors	The five patterns were interpreted using ‘Characteristic Statements of the Five Types Based on Those Ranked at the Most Positive Ends (+3, +4)’ (Table 3), thereby enabling to identify the most prominent viewpoints associated with each pattern	14–15
3.5	Statistical software	We used PQMethod 2.35 software (Schmolck and Atkinson 2014), a commonly used and validated software package for Q‐methodology studies	12
4.	Presentation findings		
4.1	Presentation findings	Table 2. Q statements and corresponding factor arrays across the five patterns. Table 3. Characteristic statements of the five types based on those ranked at the most positive ends (+3, +4)	Tables 2 and 3
4.2	Factor arrays	Table 2. Q statements and corresponding factor arrays across the five patterns	Table 2
4.3	Distinguishing and consensus statements	Distinguishing statements are indicated by asterisks (*) in Table 2. There were no consensus statements in this study	Table 2
4.4	Description of the factors	The descriptions of the five patterns were reported in Section 3	14–19

### 2.2 Study Design and Participants

The research used the Q methodology (Stephenson 1953) to investigate nurses' perceptions of TCM care for menstrual symptoms. The participating nurses arranged Q statements according to their subjective thoughts, and factor analysis was conducted to analyse and summarize their attitudes toward TCM care for menstrual symptoms.

Considering that hospital size and the resulting differences in workload may influence how nurses manage menstrual symptoms, we strategically selected one medical center and one regional hospital, with 30 nurses recruited from each institution. This strategic sampling is consistent with Q methodology, which seeks to identify the existence of distinct viewpoints rather than to generalize findings to a broader population (Watts and Stenner 2012). It is also consistent with prior guidance suggesting that a participant group of approximately 40–60 is adequate for Q‐method studies (Smith et al. 1995).

The inclusion criteria required that participants were employed at one of the hospitals, had previously used TCM treatments for menstrual symptoms, were willing to participate in the study, and had signed informed consent forms. Because prior use of TCM treatments for menstrual symptoms was a key inclusion criterion, other sampling methods may not be feasible; therefore, convenience and snowball sampling were employed. After obtaining Institutional Review Board (IRB) approval, we recruited 60 eligible nurses through advertisements posted on hospital websites and through snowball sampling.

### 2.3 Measures of Demographic Data

A questionnaire was administered to collect information on the nurses' backgrounds, including age, years of work experience, education, marital status, pregnancy history, most common type of work shift, dysmenorrhea during the last menstruation, pain intensity, use of menstrual leave and self‐purchase of painkillers. Menstrual pain intensity over the past month was assessed using a numeric rating scale (NRS 0–10), as suggested by Evans et al. (2021).

### 2.4 Q‐Set Development

The Q statements were adapted from the research team's prior study (Tsai et al. 2016). Forty women using TCM and experiencing menstrual discomfort participated in qualitative interviews exploring their lived experiences of menstrual symptoms and management strategies for menstrual health, including TCM use for symptom relief. From the qualitative data, statements were extracted concerning menstrual discomfort experiences, daily life impacts, TCM care expectations and self‐care practices. This yielded an initial pool of 134 statements, which researchers refined by consolidating similar items and eliminating duplicates, resulting in 57 statements. Five experts (nursing scholars, clinical nurses, nursing administrators and Q methodology researchers) then evaluated the content validity of the revised statements. Finally, 41 statements with content validity index (CVI) values > 0.8 were retained and categorized into five dimensions: (1) symptoms across the menstrual cycle, (2) life impacts of menstrual symptoms, (3) trust and expectations regarding TCM care efficacy, (4) factors affecting TCM application and (5) self‐care strategies for menstrual health (Table 2). All 41 Q statements were textual statements without any visual items.

**TABLE 2:** Q statements and corresponding factor arrays across the five patterns.

Category	Q statement	Item no.	Factor arrays				
			F1	F2	F3	F4	F5
A. Symptoms across the menstrual cycle							
Menstrual pain	I believe menstrual pain is a warning sign of health issues that should be taken seriously	32	1	4**	0	−1*	1
	I believe that experiencing menstrual pain is a regular occurrence, and I have become accustomed to it	1	2	−4**	0	−1*	2
	I have come to accept menstrual pain as a normal aspect of my body and no longer view it as a disease	33	−1	−3*	−1	1	1
Multiple menstrual symptoms	I am experiencing symptoms related to my menstrual cycle, such as dizziness, cold sweats, vomiting, or nausea	40	0*	1**	−3*	−4*	−2*
	I experience severe menstrual pain, but it only occurs during my periods	**2**	4*	−3**	0*	2*	3*
PMS	I am experiencing symptoms of PMS, which include weight gain, breast pain and oedema	39	2*	**3**	−3	0	**3**
	I am experiencing symptoms of PMS, including mood swings, anxiety, fatigue and irritability	41	1	**3**	−4**	1	**4**
B. Life impacts of menstrual symptoms							
Negative impacts on work and daily life	I was unable to come to work because of menstrual pain	8	2	−2**	−4	2	−4
	I didn't notice the problem until the pain started affecting my thinking, concentration and productivity	**9**	**3**	1**	−1	4*	0*
	Severe menstrual pain is affecting my daily life and causing anxiety	10	−4	−2	−2	2**	−2
	Menstrual pain often affects my ability to participate in social activities	11	1	1	0	3**	0
	I have difficulty sleeping due to menstrual pain	**12**	**3**	**3**	−2	−1	−2
Others misunderstand my menstrual discomfort	I am feeling extremely uncomfortable, but it seems that others are unable to understand the level of my distress	3	0*	−2	2*	−3	−3
	My menstrual symptoms are only seen as severe when they are physically visible (e.g., paleness, weakness, cold sweats)	4	1	1	2**	0	−2**
	Due to my dysmenorrhea, some people assume that I have poor health and workability	13	−1	−1	−2	2**	−2
	I don't want to take menstrual leave because I am afraid of being seen as lazy	14	2**	0	−1	1	0
	My religious beliefs and the companionship of my relatives and friends help me endure my menstrual pain	34	−2	−1	−2	−3	−1
C. Trust and expectation regarding the efficacy of TCM care							
Trust the effect of TCM care	I prefer TCM over painkillers for relief from menstrual pain	15	0*	0*	**4**	**4**	2**
	Physical conditioning using TCM is a crucial way to reduce menstrual pain	19	0*	1*	**3**	**3**	2
	Due to ‘neglecting my health conditioning’, I am experiencing menstrual symptoms	38	−2	0*	−3	−2	0**
Expect TCM can improve menstrual health	I am using TCM to improve my fertility	18	−4**	0*	4**	−1*	−4**
	I am hoping to find relief from menstrual pain without relying on pain medication	**17**	4**	2	1*	−1**	**3**
	I hope that TCM therapy can alleviate my menstrual pain so that it does not affect my daily life	16	2	2	3*	1	2
D. Factors affecting TCM application							
The reason for the TCM care application	I was recommended TCM treatment by my friends and relatives for relief from menstrual discomfort	20	−1**	0	1	3**	0
	I only visit a doctor when I feel unwell	27	0	0	−1	0	1**
	Even without a prescription, I have tried TCM regimens such as Si‐Wu‐Tang, Zhong‐Jiang‐Tang and Sheng‐Hua‐Tang	21	−3**	0*	1	1	2
Difficult to apply TCM	As I did not experience the expected effectiveness, I did not take the medications seriously	26	−2	−1	1*	−2	0
	Since TCM treatment did not provide me with immediate relief, I switched to painkillers for my menstrual pain	23	−1	−1	0	−1	−1
	Despite knowing that not taking my medication as prescribed will reduce its effectiveness, I still do so	25	−2	−2	0	−2	1
Concerned about TCM side effects	I am concerned about the possible negative impacts of using TCM for an extended time	24	−3**	0	0	0	−1
	I am worried about the side effects of medication, so I sometimes adjust the dosage or stop taking it	28	−2	−2	0	0	−1
E. Applying self‐care strategies for menstrual health							
Essential self‐care strategies	I have realized the importance of adopting a healthy lifestyle due to the pain I experience during menstruation	37	−1	4**	−1	1*	1*
	Regular exercise and nutritional supplements have limited benefits in improving menstrual health	6	0	−3	2**	0	−3
	Depending solely on regular exercise or nutritional supplements to improve menstrual health is not cost‐effective	7	0	−4	1**	0	−3
	It is unnecessary to consume nutritional supplements during menstruation	35	−1	0	−2*	−4*	0
Strategies of self‐care	I have tried several methods, such as hot drinks, compresses and massage, to alleviate menstrual pain; however, I have only experienced mild relief	**5**	**3**	−1**	**3**	2	**4**
	I am trying out acupressure and dietary supplements that have been recommended on the internet for menstrual health	22	−3**	−1	2*	0	−1
	I monitor my body for changes and use self‐care methods to alleviate menstrual discomfort	36	0	2**	−1	−2	0
Difficulty in applying self‐care	TCM prescriptions for lifestyle and dietary adjustments are challenging, such as avoiding late nights and cold food or drinks	29	1	1*	0	−2**	−1
	Due to my work schedule, I cannot go to bed early or on time	30	1	2	2	−3**	0
	Regular exercise can aid in reducing menstrual discomfort, but it can be challenging to sustain	31	0	2**	1	0*	1
	Eigenvalues		9.09	5.35	4.73	4.03	3.29
	% Variance		15.14	8.92	7.88	6.72	5.48

*Note:* Distinguishing statements factor * *p* < 0.05, ** *p* < 0.01. Bold type indicates both outer cores (most agree +4 and +3) of the composite sorting of each factor.

Abbreviations: PMS, premenstrual syndrome; TCM, traditional Chinese medicine.

### 2.5 Data Collection Procedure

**FIGURE 1:**
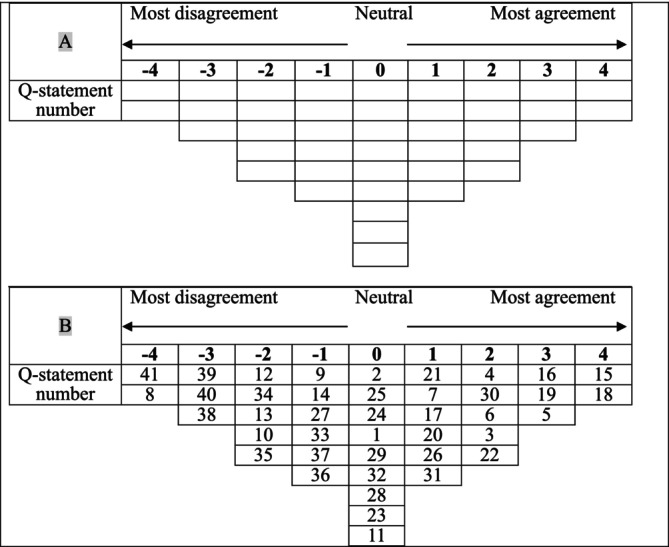
A Q‐sort grid for rank‐ordering Q statements. (A) 41 cards on the Q‐grid were sorted by participants on a 9‐point scale from −4 (most disagreement) to +4 (most agreement). (B) An idealized Q‐sort grid of Pattern 3.

This study adopted a forced‐choice distribution, which requires participants to assign a specific number of items to each ranking value. This approach aligns with Q methodology's intention to capture holistic configurations and to reveal individual viewpoints as a whole (Watts and Stenner 2012). Specifically, we chose a nine‐point (−4 to +4) forced distribution with a steep shape, placing more items near the middle to reflect participants' likely indifference toward many Q statements. This design was informed by our prior qualitative research, which revealed that women's experiences of menstrual symptoms and management strategies tend to focus on just a few most habitual approaches, even when Q statements offered a variety of strategies. The number of cells under each point followed Watts and Stenner's illustration (figure 4.1, 81, Watts and Stenner 2012): +4 (2 cells), +3 (3 cells), +2 (5 cells), +1 (6 cells), 0 (9 cells in this study; 8 cells in Watts and Stenner), with symmetrical distribution for negative values. The Q‐sort grid used in this study is shown in Figure 1.

The research team met individually with interested participants to explain the study purpose and provide written informed consent forms. Participants who signed the consent forms were then invited to complete the Q‐sort of the designed statements and answer a structured questionnaire. Participants were first guided to review the list of 41 Q statements and mark those they agreed with (+), disagreed with (−), or felt neutral toward (no mark). The research team then provided participants with laptop computers to perform the Q‐sort on the e‐platform, which featured a list of Q statements on the left and the Q‐sort grid on the right. Participants dragged statements from the left panel to the appropriate positions on the right Q‐sort grid. For example, placing those they most agreed with in the rightmost column (+4) and those they most disagreed with in the leftmost column (−4). Statements could be repositioned by dragging them within the Q‐sort grid or returned to the left‐side list if needed. The e‐platform interface was adapted from our team's previous work (Huang et al. 2020). This platform automatically exported Q‐sort data in a format compatible with PQMethod for analysis. Upon completion of the Q‐sort, participants were invited to provide brief rationales for their selections in the extreme categories (+3 to +4 and −3 to −4). Each participant received a small gift as a token of appreciation for their time and contribution.

### 2.6 Data Analysis

We used PQMethod 2.35 software (Schmolck and Atkinson 2014), a commonly used and validated software package for Q‐methodology studies, to perform factor analysis on each participant's Q‐sorting of 41 Q‐statements. Factor extraction and rotation were conducted using principal component analysis with varimax rotation, which maximizes similarities within patterns and differences between them to facilitate the identification of similar perspectives.

Eigenvalues > 1.0 were used as a criterion for retaining factors, and the number of patterns was further informed by eigenvalues and scree plots (Shin et al. 2018; Watts and Stenner 2012). In this study, we adopted a five‐factor solution, as all eigenvalues were above 1 and the scree plot slope began to level off after the fifth factor. Eigenvalues for the fifth and the sixth factor were 3.29 and 3.16, respectively. The five‐factor solution explained 44% of the total variance. As previously noted in the literature, solutions accounting for approximately 35%–40% or more of the variance are generally considered robust (Watts and Stenner 2012, 105). The appropriateness of the reported factor solution is described as follows: First, the composite reliability values for the five factors ranged from 0.96 to 0.98, exceeding commonly recommended thresholds (e.g., 0.70). This indicates strong internal consistency of the Q sorts within each factor and supports the reliability of the extracted factor structure. The composite reliability values for the five factors were 0.98, 0.98, 0.96, 0.96 and 0.97. Second, the absolute values of the factor score correlations ranged from approximately 0 to 0.30, indicating weak to modest associations between factors. This suggests that the factor solution captures reasonably distinct viewpoints without excessive overlap. In the context of Q methodology, the combination of high composite reliability and low inter‐factor correlations supports the suitability and interpretability of the factor solution.

After determining the five‐factor solution, we first applied PQMethod's automatic flagging procedure to flag each participant's Q‐sort and thereby identify the factor‐defining Q‐sorts for the five study factors. Of the 60 participants, 51 were included in the final analysis. Four Q‐sorts were excluded because their factor loadings were below 0.40 on all five factors, using the criterion derived from the formula suggested by Brown (1980). At the 0.01 significance level, significant factor loadings were defined as those with an absolute value equal to or > 2.58 divided by the square root of the number of Q‐statements. For the 41 Q‐statements used in this study, the criterion for statistically significant factor loadings at the 0.01 level was set at 2.58/√41≈0.40. Another five Q‐sorts were excluded; although one of their absolute factor loadings exceeded the 0.40 threshold, the loading itself was negative and was therefore removed manually. In Q‐methodology, factor patterns are typically interpreted as coherent viewpoints, and negative loadings represent disagreement or opposition that is more difficult to integrate into a clear interpretive narrative.

### 2.7 Ethical Considerations

This study was approved by the IRB of Taipei Veterans General Hospital (approval no. 2017‐01‐004B). The participants were invited to join voluntarily and signed an informed consent form. The participants were identified only by numbers in the Q‐sorting data to maintain anonymity.

## 3 Results

The study used Q‐factor analysis to group similar subjective viewpoints of menstrual symptoms and TCM care among nurses. The final model included data from 51 participants, accounting for 44.14% of the total variance. The five factors accounted for 15.14%, 8.92%, 7.88%, 6.72% and 5.48% of the variance, respectively (Table 2). The five patterns were interpreted using ‘Characteristic Statements of the Five Types Based on Those Ranked at the Most Positive Ends (+3, +4)’ (Table 3), thereby enabling identification of the most prominent viewpoints associated with each pattern. The distribution of demographic backgrounds across the five patterns among nurses is presented in Table 4. Demographic differences between the five patterns were compared statistically. However, no significant differences in demographic characteristics were found among the groups, as indicated by both ANOVA and chi‐square tests (all *p* > 0.05).

**TABLE 3:** Characteristic statements of the five types based on those ranked at the most positive ends (+3, +4).

Item	Q statement	Rank	*Z* score
Pattern 1: Expecting pain relief without painkillers			
17	I am hoping to find relief from menstrual pain without relying on pain medication	4	2.014
2	I experience severe menstrual pain, but it only occurs during my periods	4	1.997
9	I didn't notice the problem until the pain started affecting my thinking, concentration and productivity	3	1.674
12	I have difficulty sleeping due to menstrual pain	3	1.67
5	I have tried several methods, such as hot drinks, compresses, and massage, to alleviate menstrual pain; however, I have only experienced mild relief	3	1.669
Pattern 2: Being aware of health warnings and prioritizing a healthy lifestyle			
32	I believe menstrual pain is a warning sign of health issues that should be taken seriously	4	1.785
37	I have realized the importance of adopting a healthy lifestyle due to the pain I experience during menstruation	4	1.774
39	I am experiencing symptoms of PMS, which include weight gain, breast pain, and oedema	3	1.736
12	I have difficulty sleeping due to menstrual pain	3	1.585
41	I am experiencing symptoms of PMS, including mood swings, anxiety, fatigue, and irritability	3	1.433
Pattern 3: Trusting TCM for menstrual and reproductive health			
15	I prefer TCM over painkillers for relief from menstrual pain	4	1.939
18	I am using TCM to improve my fertility	4	1.89
16	I hope that TCM therapy can alleviate my menstrual pain so that it does not affect my daily life	3	1.583
19	Physical conditioning using TCM is a crucial way to reduce menstrual pain	3	1.518
5	I have tried several methods, such as hot drinks, compresses, and massage, to alleviate menstrual pain; however, I have only experienced mild relief	3	1.421
Pattern 4: Relying on TCM for pain management and daily activities			
15	I prefer TCM over painkillers for relief from menstrual pain	4	2.092
9	I didn't notice the problem until the pain started affecting my thinking, concentration, and productivity	4	1.737
19	Physical conditioning using TCM is a crucial way to reduce menstrual pain	3	1.683
11	Menstrual pain often affects my ability to participate in social activities	3	1.435
20	I was recommended TCM treatment by my friends and relatives for relief from menstrual discomfort	3	1.317
Pattern 5: Exploring various TCM care for valuable outcomes			
5	I have tried several methods, such as hot drinks, compresses, and massage, to alleviate menstrual pain; however, I have only experienced mild relief	4	1.79
41	I am experiencing symptoms of PMS, including mood swings, anxiety, fatigue, and irritability	4	1.551
2	I experience severe menstrual pain, but it only occurs during my periods	3	1.487
39	I am experiencing symptoms of PMS, which include weight gain, breast pain, and oedema	3	1.361
17	I am hoping to find relief from menstrual pain without relying on pain medication	3	1.343

Abbreviations: PMS, premenstrual syndrome; TCM, traditional Chinese medicine.

**TABLE 4:** Sociodemographic characteristics of nurses across the five types.

Characteristics	Total (*n* = 60)	Pattern arrays (*n* = 51)				
		Pattern 1 (*n* = 14)	Pattern 2 (*n* = 18)	Pattern 3 (*n* = 5)	Pattern 4 (*n* = 5)	Pattern 5 (*n* = 9)
		Expecting pain relief without painkillers	Being aware of health warnings and prioritizing a healthy lifestyle	Trusting TCM for menstrual and reproductive health	Relying on TCM for pain management and daily activities	Exploring various TCM care for valuable outcomes
	*n* (%) or mean ± SD	*n* (%) or mean ± SD	*n* (%) or mean ± SD	*n* (%) or mean ± SD	*n* (%) or mean ± SD	*n* (%) or mean ± SD
Age	32.88 ± 7.63	31.57 ± 7.81	33.61 ± 7.90	33.20 ± 5.22	36.00 ± 7.31	32.44 ± 8.35
Work years	9.72 ± 6.51	7.48 ± 6.03	10.37 ± 6.13	12.78 ± 4.41	10.18 ± 3.70	10.16 ± 8.78
Education						
Junior college	11 (18.33)	2 (14.29)	3 (16.67)	2 (40.00)	1 (20.00)	1 (11.11)
College	43 (71.67)	12 (85.71)	14 (77.78)	2 (40.00)	3 (60.00)	5 (55.56)
≥Graduate school	6 (10.00)	0 (0.0)	1 (5.56)	1 (20.00)	1 (20.00)	3 (33.33)
Marital status						
Single	39 (65.0)	10 (71.43)	11 (61.11)	3 (60.0)	3 (60.0)	7 (77.78)
Married	21 (35.0)	4 (28.57)	7 (38.89)	2 (40.0)	2 (40.0)	2 (22.22)
Pregnancy history						
No	41 (68.33)	10 (71.43)	15 (83.33)	2 (40.00)	2 (40.00)	7 (77.78)
Yes	19 (31.67)	4 (28.57)	3 (16.67)	3 (60.00)	3 (60.00)	2 (22.22)
Most common types of work shift						
Rotating shifts	19 (31.67)	5 (35.71)	5 (27.78)	1 (20.00)	2 (40.00)	1 (11.11)
Day shifts	17 (28.33)	3 (21.43)	4 (22.22)	1 (20.00)	0 (0.0)	6 (66.67)
Evening shifts	16 (26.67)	3 (21.43)	7 (38.89)	3 (60.00)	2 (40.00)	1 (11.11)
Night shifts	8 (13.33)	3 (21.43)	2 (11.11)	0 (0.0)	1 (20.00)	1 (11.11)
Dysmenorrhea during the last menstruation	37 (61.67)	7 (50.00)	11 (61.11)	3 (60.00)	4 (80.00)	5 (55.56)
Pain intensity	5.87 ± 2.28	7.21 ± 1.63	5.56 ± 2.64	6.80 ± 1.64	5.20 ± 2.49	4.89 ± 2.37
Use of menstrual leave						
No	25 (41.67)	4 (28.57)	6 (33.33)	3 (60.00)	2 (40.00)	5 (55.56)
Yes	16 (26.67)	3 (21.43)	6 (33.33)	1 (20.00)	2 (40.00)	0 (0.0)
Desired	19 (31.67)	7 (50.00)	6 (33.33)	1 (20.00)	1 (20.00)	4 (44.44)
Taking self‐purchased painkillers						
No	13 (21.67)	2 (14.29)	4 (22.22)	1 (20.00)	2 (40.00)	3 (33.33)
Yes	47 (78.33)	12 (85.71)	14 (77.78)	4 (80.00)	3 (60.00)	6 (66.67)

### 3.1 Pattern 1: Expecting Pain Relief Without Painkillers

Fourteen participants reported experiencing significant menstrual symptoms, specifically menstrual pain, which had a substantial impact on their lives. As shown in Table 3, they rated their pain as severe during periods (+4) and stated that it affected their ability to sleep during their menstrual period (+3). The participants reported that they did not face any problems until the pain began to affect their thinking, concentration and ability to work efficiently (+3). They expressed a desire for ways to ease their menstrual pain without using pain medication (+4). As a result, they have tried various treatments, including herbal medicines, hot drinks, hot packs, folk remedies and oil massages. However, they reported that these treatments have limited effectiveness (+3).

Based on the factor array, statements statistically distinguishing Pattern 1 (*p* < 0.01) were as follows (Appendix S1). Positively endorsed statements were ‘I am hoping to find relief from menstrual pain without relying on pain medication’ (+4) and ‘I don't want to take menstrual leave because I am afraid of being seen as lazy’ (+2). Negatively endorsed statements were ‘I am using TCM to improve my fertility’ (−4), ‘Even without a prescription, I have tried TCM regimens such as Si‐Wu‐Tang, Zhong‐Jiang‐Tang, and Sheng‐Hua‐Tang’ (−3), ‘I am trying out acupressure and dietary supplements that have been recommended on the internet for menstrual health’ (−3), ‘I am concerned about the possible negative impacts of using TCM for an extended time’ (−3) and ‘I was recommended TCM treatment by my friends and relatives for relief from menstrual discomfort’ (−1). Overall, participants in Factor 1 were concerned with avoiding pain medication and menstrual leave, and showed less interest in TCM.

Compared with the participants in the other patterns, the nurses associated with Pattern 1 were relatively younger, with an average age of 31.57 years, and had less clinical work experience, with an average of 7.48 years. Most had a college education (85.71%) and had never been pregnant (71.43%). Relatively, nurses belonging to Pattern 1 had the highest pain score (NRS = 7.21), the highest rate of taking self‐purchased painkillers (85.71%), and 50% of them desired menstrual leave.

### 3.2 Pattern 2: Being Aware of Health Warnings and Prioritizing a Healthy Lifestyle

Eighteen participants presented significant symptoms related to menstrual pain. As can be seen in Table 3, they reported having trouble sleeping due to menstrual pain (+3), as well as premenstrual symptoms such as weight gain, breast pain, oedema (+3), emotional instability, anxiety, tiredness and anger (+3). These participants recognized the seriousness of their menstrual pain as a health warning (+4) and the importance of adopting a healthy lifestyle (+4).

Based on the factor array, statements that statistically distinguish Pattern 2 (*p* < 0.01) were as follows (Appendix S2). Positively endorsed statements were ‘I believe menstrual pain is a warning sign of health issues that should be taken seriously’ (+4), ‘I have realized the importance of adopting a healthy lifestyle due to the pain I experience during menstruation’ (+4), ‘Regular exercise can aid in reducing menstrual discomfort, but it can be challenging to sustain’ (+2), ‘I monitor my body for changes and use self‐care methods to alleviate menstrual discomfort’ (+2), ‘I didn't notice the problem until the pain started affecting my thinking, concentration and productivity’ (+1), ‘I am experiencing symptoms related to my menstrual cycle, such as dizziness, cold sweats, vomiting, or nausea’ (+1), and ‘Physical conditioning using TCM is a crucial way to reduce menstrual pain’ (+1). Negatively endorsed statements were ‘I believe that experiencing menstrual pain is a regular occurrence, and I have become accustomed to it’ (−4), ‘I experience severe menstrual pain, but it only occurs during my periods’ (−3), ‘I was unable to come to work because of menstrual pain’ (−2), and ‘I have tried several methods, such as hot drinks, compresses, and massage, to alleviate menstrual pain; however, I have only experienced mild relief’ (−1). In summary, participants in Factor 2 were concerned with recognizing menstrual pain as a serious health issue and adopting healthier self‐care habits, and demonstrated less acceptance of menstrual pain as a normal occurrence.

The average age of the nurses who loaded onto Pattern 2 was 33.61 years, with an average duration of clinical work experience of 10.37 years. A total of 83.33% had an education level equivalent to a bachelor's degree or above. Most were single (61.11%) and had never been pregnant (83.33%). Furthermore, these nurses reported a moderate pain score (NRS = 5.56), with 77.78% taking self‐purchased painkillers and 33.33% desiring menstrual leave.

### 3.3 Pattern 3: Trusting TCM for Menstrual and Reproductive Health

Five participants' Q‐sorts were significantly loading on Pattern 3. They showed significant results in trying TCM to improve fertility (+4). They preferred not to rely on painkillers and believed that TCM care was the appropriate approach (+4). According to them, TCM practices were the fundamental way to treat menstrual pain (+3). They also hoped that TCM care would help relieve their menstrual pain and not affect their daily life (+3). Despite various treatments, such as hot drinks, compresses and massages, they have shown limited effectiveness in relieving menstrual pain (+3). The results can be seen in Table 3.

Based on the factor array, statements that statistically distinguish Pattern 3 (*p* < 0.01) were as follows (Appendix S3). Positively endorsed statements were ‘I am using TCM to improve my fertility’ (+4), ‘My menstrual symptoms are only seen as severe when they are physically visible (e.g., paleness, weakness, cold sweats)’ (+2), ‘Regular exercise and nutritional supplements have limited benefits in improving menstrual health’ (+2), ‘Depending solely on regular exercise or nutritional supplements to improve menstrual health is not cost‐effective’ (+1). Negatively endorsed statement was ‘I am experiencing symptoms of PMS, including mood swings, anxiety, fatigue and irritability’ (−4). Overall, participants in Factor 3 were concerned with using TCM to improve fertility and indicated that menstrual symptoms were only recognized as a serious problem when they became physically visible, while placing less emphasis on PMS‐related symptoms.

The Pattern 3 nurses consisted of five participants with an average age of 33.2 years. They have extensive clinical work experience, averaging approximately 12.78 years. Eighty percent had fixed work shifts, and 60% did not request menstrual leave. Additionally, 80% of these nurses reported an average pain score of 6.8 on the NRS, and they relied on self‐purchased painkillers.

### 3.4 Pattern 4: Relying on TCM for Pain Management and Daily Activities

Five participants' Q‐sorts were significantly loading on Pattern 4. They all agreed that menstrual pain hurts their ability to think, concentrate, work efficiently (+4), and participate in social activities (+3). The nurses in Pattern 4 were consistent with those in Pattern 3, as they expressed hesitation toward using painkillers for relief from menstrual pain. Instead, they preferred TCM, as it involves physical conditioning, which they believe is a better way to alleviate menstrual pain (+4). They also mentioned that TCM treatments have been effective for their friends and family, so they recommend them to them (+3) (Table 3).

Based on the factor array, statements that statistically distinguish Pattern 4 (*p* < 0.01) were as follows (Appendix S4). Positively endorsed statements were ‘Menstrual pain often affects my ability to participate in social activities’ (+3), ‘I was recommended TCM treatment by my friends and relatives for relief from menstrual discomfort’ (+3), ‘Severe menstrual pain is affecting my daily life and causing anxiety’ (+2), and ‘Due to my dysmenorrhea, some people assume that I have poor health and workability’ (+2). Negatively endorsed statements were ‘Due to my work schedule, I cannot go to bed early or on time’ (−3), ‘TCM prescriptions for lifestyle and dietary adjustments are challenging, such as avoiding late nights and cold food or drinks’ (−2), and ‘I am hoping to find relief from menstrual pain without relying on pain medication’ (−1). Collectively, participants in Factor 4 were concerned with the social and daily‐life impact of menstrual pain, and demonstrated less concern about TCM‐related lifestyle restrictions.

The nurses who were part of Pattern 4 had an average age of 36 years and an average clinical work experience of 10.18 years. Most had an education level equivalent to a bachelor's degree or above (80%), 40% were married and 60% had experienced pregnancy (Table 3). Their pain score on the NRS was 5.2, and 60% used self‐purchased painkillers (Table 3).

### 3.5 Pattern 5: Exploring Various Types of TCM Care for Valuable Outcomes

In the present study, nine nurses were identified with Pattern 5. They all shared similar experiences of suffering from various premenstrual symptoms, such as weight gain, breast pain, oedema (+3), mood swings, anxiety, tiredness, and anger (+4). Although they only experienced these symptoms during their menstrual period (+3), they reported severe menstrual pain. They expressed a desire for pain relief without depending on medication (+3). They had tried treatments such as herbal medicines, hot drinks, hot packs, folk remedies, and oil massages, but with limited effectiveness (+4) (Table 3).

Based on the factor array, statements that statistically distinguish Pattern 5 (*p* < 0.01) were as follows (Appendix S5). Positively endorsed statements were ‘I prefer TCM over painkillers for relief from menstrual pain’ (+2), and ‘I only visit a doctor when I feel unwell’ (+1). Negatively endorsed statements were ‘I am using TCM to improve my fertility’ (−4), and ‘My menstrual symptoms are only seen as severe when they are physically visible (e.g., paleness, weakness, cold sweats)’ (−2). One neutrally ranked statement (0), which was ‘Due to neglecting my health conditioning, I am experiencing menstrual symptoms’. In summary, participants in Factor 5 were concerned with preferring TCM over painkillers and seeking medical care only when feeling unwell, and showed less interest in using TCM to improve fertility.

In Pattern 5, the average age of the participants was 32.44 years. 88.89% of them hold an education level equivalent to an undergraduate degree or above. Two‐thirds (66.67%) worked fixed day shifts, whereas 77.78% were single and had never been pregnant. They experienced some degree of menstrual pain, with an average NRS score of 4.89, as shown in Table 3.

## 4 Discussion

We aimed to use Q methodology to explore the unique perspectives of nurses experiencing menstrual symptoms within the occupational context. Q factor analysis identified five distinct patterns among nurses with varying approaches to menstrual symptom management and perceptions of TCM care. The following section provides a detailed discussion of the study findings.

Among nurses in Pattern 1, who reported an average pain score of 7.21 on a 1–10 scale, over 85% resorted to self‐medication with painkillers. The prevalence of dysmenorrhea among hospital nurses has been reported to be 69.9%, with 86.0% experiencing moderate to severe symptoms (Yöndem and Çıtak Bilgin 2020). Interestingly, despite their frequent use of analgesics, nurses in Pattern 1 expressed a strong preference for achieving pain relief without relying on medication. This finding highlights a significant dilemma in managing menstrual discomfort. Although nurses seek effective pain relief, their reluctance to use analgesics may reflect concerns about dependency or potential side effects (Dassieu et al. 2022).

In this study, 61.67% of nurses were experiencing dysmenorrhea during their last menstruation. In addition, this study revealed that approximately 78% of nurses self‐medicate with painkillers to manage menstrual symptoms, highlighting the widespread reliance on pharmacological solutions for pain relief. Nurses, as professional healthcare providers, despite their advanced knowledge, face the same challenges and dilemmas as the general population when managing chronic or recurrent pain. Studies have shown that dilemmas and discrepancies often arise between patients and pharmacists when deciding whether to prioritize pain relief or prevent adverse effects (Dassieu et al. 2022).

Descriptively, Pattern 1 included relatively younger nurses with fewer years of clinical experience (7.48 years; compared to a range of 7.48–12.78 years across the five patterns) and the relatively high proportion expressing a desire for menstrual leave (50%). Although these differences were not statistically significant, they may still suggest that nurses in this group experience a greater immediate symptom burden relative to their work demands. From a practical perspective, this pattern underscores the importance of providing workplace‐based education on appropriate analgesic use as well as feasible nonpharmacological pain management strategies for nurses with recurrent menstrual discomfort (Goucke et al. 2015).

Pattern 2 nurses recognized menstrual symptoms as health warnings and expressed a desire to self‐manage them through a healthy lifestyle. However, they may face significant challenges in adopting such practices due to their rotating schedules and night shifts (Yöndem and Çıtak Bilgin 2020). Shift work disrupts routines, making it difficult to maintain a healthy lifestyle to improve menstrual health. This finding further suggests that improving menstrual health among nurses cannot rely on individual health awareness alone.

Pattern 2 descriptively included a relatively high proportion of evening shifts (38.89%) and more than half of nurses who experienced dysmenorrhea during their recent menstruation (61.11%). These tendencies may provide additional context for understanding why this group emphasized menstrual symptoms as warning signs and highlighted lifestyle adjustment. Occupational characteristics and physical work demands have also been associated with dysmenorrhea among nurses, highlighting the potential value of work environment improvement in reducing symptom burden (Jung et al. 2024). Therefore, institutional and occupational health policies that support sleep, recovery, and more sustainable work routines may be equally important.

In this study, nurses who adhered to Patterns 3 and 4 tended to favour TCM to address menstrual symptoms. Previous research has shown that most women prefer self‐care strategies for managing menstrual symptoms, often relying on home remedies or lifestyle adjustments rather than seeking formal medical care (Armour, Parry, Manohar, et al. 2019; Tsonis et al. 2021). TCM care is widely trusted for treating dysmenorrhea because it focuses on altering the body's constitution through diet and lifestyle modifications, rather than solely relying on medication (Harvie et al. 2019). Nurses of Pattern 3 expressed interest not only in relieving menstrual pain but also in improving fertility and perceived TCM as a potential solution. Similar findings have been reported in studies of women of reproductive age (Tsai et al. 2016). Descriptive tendencies of Pattern 3 may provide additional context for interpretation. This pattern included nurses with the longest average clinical experience (12.78 years; compared to a range of 7.48–12.78 years across the five patterns) and a relatively high proportion working evening shifts (60%). Prolonged exposure to shift work, particularly evening or night shifts, may influence menstrual health over time. This context may help explain why some participants, particularly in Pattern 3, associated TCM with both menstrual pain relief and fertility‐related benefits. These tendencies may suggest that trust in TCM for menstrual and reproductive health (Tsai et al. 2016) is shaped by concerns that extend beyond immediate symptom relief, encompassing broader considerations such as long‐term reproductive health and fertility‐related expectations.

Among the nurses in Pattern 4 who reported less severe pain (average score of 5.20 on a 1–10 scale), many acknowledged how menstrual pain subtly affected their work and social life. Initially, they did not recognize the symptoms as problematic until the pain began interfering with their daily activities. They believe that TCM can alleviate menstrual discomfort, and their family and friends also support the use of TCM to manage these symptoms. The nurses in Pattern 4, despite the apparently less severe pain, found these discomfort symptoms gradually became a source of distress. The progressive impact on daily functioning highlights that even moderate menstrual symptoms can significantly affect well‐being. The interpretation of these findings should take culturally sensitive menstrual care into account. Previous research supports TCM as a therapeutic option for the management of menstrual symptoms (Chiou et al. 2020). Survey data indicate that people have used TCM and hold positive expectations of it for daily health maintenance and disease prevention (Chuang et al. 2025). Nurses' trust in TCM may reflect not only individual treatment preference but also broader beliefs about bodily regulation, physical conditioning, and health practices.

Pattern 4 included the oldest subgroup on average (36.00 years; compared to a range of 31.57–36.00 years across the patterns) and a relatively high proportion of nurses who experienced dysmenorrhea during their recent menstruation (80%). From a practical perspective, this pattern suggests that trust in TCM may not be based solely on immediate symptom relief, but also on accumulated health concerns shaped by age and repeated symptom experience. Some nurses may view TCM as part of a broader strategy for maintaining overall health.

With relatively less severe pain (average score of 4.89 on a 1–10 scale), Pattern 5 nurses still reported experiencing both physical and psychological discomfort during their menstrual cycle. They tried various self‐care management strategies but found the results to be limited. The findings suggested that the use of self‐care or TCM‐related approaches does not necessarily indicate strong confidence in their effectiveness. Rather, it may reflect the subjective and fluctuating nature of menstrual discomfort, as well as the limited availability of consistently effective management options. Women often experiment with multiple strategies in an attempt to alleviate symptoms, even when the benefits are uncertain (Armour, Parry, Al‐Dabbas, et al. 2019). This process of trying different strategies may also be accompanied by the frustration of feeling that their efforts to manage symptoms are in vain.

Descriptively, Pattern 5 had the highest proportions of nurses with graduate‐level education (33.33%) and of those working day shifts (66.67%). Although these characteristics were not statistically significant, they may suggest that the exploration of multiple TCM‐related strategies was not limited to nurses with the most severe pain but also occurred among those trying to manage recurring discomfort in everyday life.

Although TCM is culturally familiar in Taiwan, nurses' use and perceptions of TCM remain heterogeneous and are shaped by individual considerations of appropriateness and personal health needs. Among the study results, two groups (Patterns 3 and 4) expressed firm trust in TCM, suggesting that it could effectively alleviate dysmenorrhea and contribute to fertility‐related benefits. In contrast, the other three groups (Patterns 1, 2 and 5) utilized TCM but did not explicitly express confidence in its effectiveness.

In Taiwan, where TCM is culturally embedded and structurally integrated into the healthcare system, nurses may be more likely to interpret menstrual symptom management through concepts such as bodily regulation and physical conditioning (Lin et al. 2019). By contrast, in many non‐East Asian settings, complementary or herbal therapies for menstrual symptoms are more often used as self‐initiated, supplementary, or alternative approaches rather than being integrated into routine health management (Fisher et al. 2016; Jiao et al. 2022). This distinction highlights how healthcare infrastructure and cultural familiarity shape the perceptions and use of TCM. The broader implication of this study is that menstrual symptom management is context‐dependent, influenced by local cultural beliefs, healthcare accessibility, and workplace environments. These factors should be considered when developing culturally responsive support strategies for nurses (Saleh et al. 2025). This cross‐context difference may help explain why the TCM‐based viewpoints identified in this study, especially in Patterns 3 and 4, are particularly salient in Taiwan, where such views may be understood as culturally coherent ways of interpreting menstrual and reproductive health. Prior research suggests that Chinese women's menstrual symptom management is shaped by culturally embedded understandings of TCM and its distinction from Western medicine, and that women receiving TCM for menstrual symptoms may hold distinct expectations (Ried 2015; Wu 2024).

### 4.1 Implications for Practice

For healthcare professionals like nurses, whose roles demand physical and mental resilience, unaddressed menstrual pain can have profound implications for their well‐being and work performance. Nurses experiencing menstrual symptoms reported that their physical and mental conditions often do not meet the demands of daily work. Despite the availability of menstrual leave, only approximately 27% of nurses in this study had ever utilized it. Factors such as nursing staff shortages and a strong sense of accountability often discourage nurses from taking menstrual leave, resulting in presenteeism. Presenteeism, where employees attend work despite not being fully functional, is a recognized workplace hazard that may exacerbate burnout (Lui et al. 2018; Rainbow and Steege 2017). Previous studies have shown that dysmenorrhea and related self‐care demands are common among hospital nurses, and that many nurses rely on analgesics to continue working despite menstrual discomfort (Chiu et al. 2013). Menstrual symptoms have also been linked to intentions to leave the workplace (Ota et al. 2023). These findings highlight the importance of supportive workplace environments, suggesting implications not only for individual well‐being but also for workforce stability and retention.

Pattern 1 nurses, despite their professional expertise, shared common concerns about medication dependency when managing menstrual pain. Promoting education on proper medication use alongside nonpharmacological pain management strategies would be helpful. Pattern 2 nurses, whose rotating schedules hinder their ability to maintain healthy lifestyles, would benefit from workplace policies that provide nutrition counselling or access to exercise facilities, enabling them to adopt healthier routines. Pattern 3 nurses expressed interest in TCM for managing menstrual pain and improving fertility, suggesting that healthcare organizations collaborate with licensed TCM practitioners to offer workshops or consultations. For Pattern 4 nurses, whose less severe symptoms subtly disrupted their work and social life, early recognition and management education could help mitigate these impacts. At last, Pattern 5 nurses, who felt frustrated by the limited success of self‐care strategies, would benefit from symptom‐tracking tools, such as apps, to identify and optimize effective management approaches.

### 4.2 Contribution of the Methodology

This study makes a methodological contribution by demonstrating the utility of Q methodology in exploring menstrual health among nurses and their lived experiences of TCM use within the caregiving‐self‐care nexus. Conventional quantitative approaches can effectively identify prevalence, severity, or treatment utilization patterns but are limited in revealing how diverse beliefs and priorities cluster within the same professional cohort. Purely qualitative methods, conversely, yield rich descriptive insights yet often fail to delineate structured patterns of shared viewpoints across participants. By employing Q methodology, this study identified five distinct subjective viewpoints concerning menstrual symptoms, self‐care practices, and TCM application. Thus, the methodological contribution of the study lies not only in describing what nurses do but also in clarifying how different forms of self‐care orientation coexist within the same occupational group. This underscores Q methodology's value for nursing research addressing complex, subjective, and shared perspectives, where the identification of patterned subjectivity is itself an important finding (Brown 1993; Ramlo 2020; Watts and Stenner 2012).

### 4.3 Limitations

This study has several limitations. First, Q methodology employs a purposive sampling approach, which limits the generalizability of the findings to a broader population. Second, the Q statements used in this study may not have captured all possible perspectives, as it is not feasible to include every viewpoint within the scope of one research. Finally, the focus on the context of TCM may not fully represent all managing strategies for menstrual symptoms. Finally, while the Q‐set was grounded in previous qualitative research (Tsai et al. 2016) and refined through expert review, it was not pretested with a separate sample of participants. This may have constrained the identification of potential issues in statement clarity or practical implementation of the Q‐sorting procedure. Future studies are therefore encouraged to incorporate and report a dedicated pilot phase to further enhance the rigour and transparency of Q methodology applications.

## 5 Conclusion

This study explored the perspectives of nurses in Taiwan who use TCM to manage menstrual symptoms. The final factor analysis model identified five distinct patterns, accounting for 44.14% of the total variance. The results indicate that nurses' responses to menstrual symptoms were not uniform but varied according to differing levels of symptom burden, work‐related disruption, expectations of self‐care, and beliefs about the role of TCM. These findings highlight the importance of workplace environments that provide flexible scheduling, access to menstrual leave, and adequate medical support, enabling nurses to adopt self‐care strategies that align with their individual needs and preferences.

## Author Contributions

S.‐H.H. and C.‐M.H. conceptualized the study design, contributed to the interpretation of data and drafted the manuscript. S.‐C.Y., S.‐F.C. and Y.‐C.C. contributed to participant recruitment and data collection. S.‐C.Y. and H.‐P.H. contributed to statistical analysis. All authors reviewed the manuscript and revised it critically for important intellectual content.

## Funding

This work was supported by a grant from the Taiwan Nurses Association (TWNA‐1062041).

## Ethics Statement

The study was approved by Taipei Veterans General Hospital's IRB (2017‐01‐004B). Participants provided info, consented voluntarily, and were identified by numbers for anonymity.

## Conflicts of Interest

The authors declare no conflicts of interest.

## Supporting information


**Appendix S1:** Characteristic and statistically distinguishing statements for pattern 1.
**Appendix S2:** Characteristic and statistically distinguishing statements for pattern 2.
**Appendix S3:** Characteristic and statistically distinguishing statements for pattern 3.
**Appendix S4:** Characteristic and statistically distinguishing statements for pattern 4.
**Appendix S5:** Characteristic and statistically distinguishing statements for pattern 5.

## Data Availability

The data that support the findings of this study are available on request from the corresponding author. The data are not publicly available due to privacy or ethical restrictions.

## References

[nop270644-bib-0001] Armour, M. , K. Parry , M. A. Al‐Dabbas , et al. 2019. “Self‐Care Strategies and Sources of Knowledge on Menstruation in 12,526 Young Women With Dysmenorrhea: A Systematic Review and Meta‐Analysis.” PLoS One 14, no. 7: e0220103. 10.1371/journal.pone.0220103. 31339951 PMC6655766

[nop270644-bib-0002] Armour, M. , K. Parry , N. Manohar , et al. 2019. “The Prevalence and Academic Impact of Dysmenorrhea in 21,573 Young Women: A Systematic Review and Meta‐Analysis.” Journal of Women's Health 28, no. 8: 1161–1171. 10.1089/jwh.2018.7615. 31170024

[nop270644-bib-0003] Brown, S. R. 1980. Political Subjectivity: Applications of Q Methodology in Political Science. Yale University Press.

[nop270644-bib-0004] Brown, S. R. 1993. “A Primer on Q Methodology.” Operant Subjectivity 16, no. 3/4: 91–138.

[nop270644-bib-0005] Bruinvels, G. , E. Goldsmith , R. Blagrove , et al. 2021. “Prevalence and Frequency of Menstrual Cycle Symptoms Are Associated With Availability to Train and Compete: A Study of 6812 Exercising Women Recruited Using the Strava Exercise App.” British Journal of Sports Medicine 55, no. 8: 438–443. 10.1136/bjsports-2020-102792. 33199360

[nop270644-bib-0006] Buchholtz, N. , and M. Vollstedt . 2024. “Q Methodology as an Integrative Approach: Bridging Quantitative and Qualitative Insights in a Mixed Methods Study on Mathematics Teachers' Beliefs.” Frontiers in Psychology 15: 1418040. 10.3389/fpsyg.2024.1418040. 39070582 PMC11273228

[nop270644-bib-0007] Chen, B. , S. Liu , F. Jin , et al. 2024. “Efficacy of Acupuncture‐Related Therapy in the Treatment of Primary Dysmenorrhea: A Network Meta‐Analysis of Randomized Controlled Trials.” Heliyon 10, no. 10: e30912. 10.1016/j.heliyon.2024.e30912. 38770299 PMC11103538

[nop270644-bib-0008] Cheng, S. Y. , P. C. Lin , Y. K. Chang , Y. K. Lin , P. H. Lee , and S. R. Chen . 2019. “Sleep Quality Mediates the Relationship Between Work–Family Conflicts and the Self‐Perceived Health Status Among Hospital Nurses.” Journal of Nursing Management 27, no. 2: 381–387. 10.1111/jonm.12694. 30328175

[nop270644-bib-0009] Chiou, S.‐J. , P.‐C. Lee , L.‐H. Lee , and K.‐C. Lin . 2020. “The Importance of Patient's Experience and Its Impact on Health Care System for People Receiving Traditional Chinese Medicine.” Journal of Alternative and Complementary Medicine 26, no. 12: 1151–1158. 10.1089/acm.2020.0143. 32945685

[nop270644-bib-0010] Chiu, M.‐H. , H.‐H. Wang , S.‐C. Hsu , and I. P. Liu . 2013. “Dysmenorrhoea and Self‐Care Behaviours Among Hospital Nurses: A Questionnaire Survey.” Journal of Clinical Nursing 22, no. 21–22: 3130–3140. 10.1111/jocn.12240. 23714141

[nop270644-bib-0011] Chuang, C.‐Y. , H.‐Y. Chung , F.‐S. Chen , and I. Arai . 2025. “A Survey of Attitudes Toward and Experiences With Traditional Chinese Medicines Among People in Taiwan.” Journal of the Chinese Medical Association 88, no. 1: 34–42. 10.1097/JCMA.0000000000001181. 39428572 PMC12718997

[nop270644-bib-0012] Dassieu, L. , E. Paul‐Savoie , É. Develay , et al. 2022. “Swallowing the Pill of Adverse Effects: A Qualitative Study of Patients' and Pharmacists' Experiences and Decision‐Making Regarding the Adverse Effects of Chronic Pain Medications.” Health Expectations 25, no. 1: 394–407. 10.1111/hex.13399. 34935258 PMC8849270

[nop270644-bib-0013] Dieteren, C. M. , N. J. S. Patty , V. T. Reckers‐Droog , and J. van Exel . 2023. “Methodological Choices in Applications of Q Methodology: A Systematic Literature Review.” Social Sciences & Humanities Open 7, no. 1: 100404. 10.1016/j.ssaho.2023.100404.

[nop270644-bib-0049] Evans, S. , C. Dowding , L. Olive , et al. 2021. “Pain Catastrophizing, but not Mental Health or Social Support, is Associated With Menstrual Pain Severity in Women With Dysmenorrhea: A Cross‐Sectional Survey.” Psychology, Health & Medicine 27, no. 6: 1410–1420. 10.1080/13548506.2021.1948581. 34190659

[nop270644-bib-0014] Fisher, C. , D. Sibbritt , L. Hickman , and J. Adams . 2016. “A Critical Review of Complementary and Alternative Medicine Use by Women With Cyclic Perimenstrual Pain and Discomfort: A Focus Upon Prevalence, Patterns and Applications of Use and Users' Motivations, Information Seeking and Self‐Perceived Efficacy.” Acta Obstetricia et Gynecologica Scandinavica 95, no. 8: 861–871. 10.1111/aogs.12921. 27185060

[nop270644-bib-0015] Goucke, C. R. , T. Jackson , W. Morriss , and J. Royle . 2015. “Essential Pain Management: An Educational Program for Health Care Workers.” World Journal of Surgery 39, no. 4: 865–870. 10.1007/s00268-014-2635-7. 24841803

[nop270644-bib-0016] Harvie, A. , A. Steel , and J. Wardle . 2019. “Traditional Chinese Medicine Self‐Care and Lifestyle Medicine Outside of Asia: A Systematic Literature Review.” Journal of Alternative and Complementary Medicine 25, no. 8: 789–808. 10.1089/acm.2018.05. 31274332

[nop270644-bib-0017] Hensel, D. , C. Toronto , J. Lawless , and J. Burgess . 2022. “A Scoping Review of Q Methodology Nursing Education Studies.” Nurse Education Today 109: 105220. 10.1016/j.nedt.2021.105220. 34902708

[nop270644-bib-0018] Huang, C. M. , J. Y. Liao , H. P. Hsu , C. Y. Lin , and J. L. Guo . 2020. “Perspectives Emerged From Students and Supervisory Staff Interaction in Drug Use Prevention: A Q Methodology Investigation.” International Journal of Environmental Research and Public Health 17, no. 15: 5621. 10.3390/ijerph17155621. 32759842 PMC7432898

[nop270644-bib-0019] Jiao, M. , X. Liu , Y. Ren , et al. 2022. “Comparison of Herbal Medicines Used for Women's Menstruation Diseases in Different Areas of the World.” Frontiers in Pharmacology 12: 751207. 10.3389/fphar.2021.751207. 35185533 PMC8854496

[nop270644-bib-0021] Jung, H. , H. Dan , C. Cha , and Y. Pang . 2024. “Dysmenorrhea and Occupational Factors.” Journal of Nursing Management 2024, no. 1: 1968522. 10.1155/jonm/1968522. 40224887 PMC11925264

[nop270644-bib-0022] Kho, K. A. , and J. K. Shields . 2020. “Diagnosis and Management of Primary Dysmenorrhea.” JAMA 323, no. 3: 268–269. 10.1001/jama.2019.16921. 31855238

[nop270644-bib-0023] Ladan, M. A. , H. Wharrad , and R. Windle . 2018. “Towards Understanding Healthcare Professionals' Adoption and Use of Technologies in Clinical Practice: Using Q‐Methodology and Models of Technology Acceptance.” BMJ Health & Care Informatics 25, no. 1: 27–37. 10.14236/jhi.v25i1.965. 29717952

[nop270644-bib-0024] Lin, P.‐Y. , C.‐H. Chu , F.‐Y. Chang , Y.‐W. Huang , H.‐J. Tsai , and T.‐C. Yao . 2019. “Trends and Prescription Patterns of Traditional Chinese Medicine Use Among Subjects With Allergic Diseases: A Nationwide Population‐Based Study.” World Allergy Organization Journal 12, no. 2: 100001. 10.1016/j.waojou.2018.11.001. 30937136 PMC6439402

[nop270644-bib-0020] Lui, J. N. M. , E. B. Andres , and J. M. Johnston . 2018. “Presenteeism Exposures and Outcomes Amongst Hospital Doctors and Nurses: A Systematic Review.” BMC Health Services Research 18, no. 1: 985. 10.1186/s12913-018-3789-z. 30567547 PMC6299953

[nop270644-bib-0025] Ota, Y. , K. Nomura , J. Hirayama , et al. 2023. “Relationship Between Somatic Symptoms With Menstruation and Intention to Leave Work Among University Hospital Nurses in Japan: A Cross‐Sectional Study.” International Archives of Occupational and Environmental Health 96, no. 1: 155–166. 10.1007/s00420-022-01905-0. 35913561

[nop270644-bib-0026] Park, J. H. , M. J. Park , and H. Y. Hwang . 2019. “Intention to Leave Among Staff Nurses in Small‐ and Medium‐Sized Hospitals.” Journal of Clinical Nursing 28, no. 9–10: 1856–1867. 10.1111/jocn.14802. 30667587

[nop270644-bib-0027] Rainbow, J. G. , and L. M. Steege . 2017. “Presenteeism in Nursing: An Evolutionary Concept Analysis.” Nursing Outlook 65, no. 5: 615–623. 10.1016/j.outlook.2017.03.005. 28416202

[nop270644-bib-0028] Ramlo, S. E. 2020. “Divergent Viewpoints About the Statistical Stage of a Mixed Method: Qualitative Versus Quantitative Orientations.” International Journal of Research & Method in Education 43, no. 1: 93–111. 10.1080/1743727X.2019.1626365.

[nop270644-bib-0029] Ray, E. , J. A. Maybin , and J. C. Harper . 2023. “Perimenopausal Women's Voices: How Does Their Period at the End of Reproductive Life Affect Wellbeing?” Post Reproductive Health 29, no. 4: 201–221. 10.1177/20533691231216162. 37984554 PMC10704889

[nop270644-bib-0030] Ried, K. 2015. “Chinese Herbal Medicine for Female Infertility: An Updated Meta‐Analysis.” Complementary Therapies in Medicine 23, no. 1: 116–128. 10.1016/j.ctim.2014.12.004. 25637159

[nop270644-bib-0031] Saleh, C. , A. Hawkey , and M. Armour . 2025. “Perceptions and Experiences of Menstrual Pain Among Middle Eastern Women Living in Australia.” Culture, Health & Sexuality: 1–12. 10.1080/13691058.2025.2573415. 41186338

[nop270644-bib-0032] Schmolck, P. , and J. Atkinson . 2014. “PQMethod Manual.”

[nop270644-bib-0050] Schoep, M. E. , T. E. Nieboer , M. van der Zanden , D. D. Braat , and A. W. Nap . 2019. “The Impact of Menstrual Symptoms on Everyday Life: A Survey Among 42,879 Women.” American Journal of Obstetrics and Gynecology 220, no. 6: 569. 10.1016/j.ajog.2019.02.04. 30885768

[nop270644-bib-0033] Scott, L. , E. Dolan , N. Baker , and Y. Melia . 2023. “Exploring Attitudes of Healthcare Professionals Towards Those With Fibromyalgia: A Q‐Methodological Approach.” British Journal of Pain 17, no. 4: 352–365. 10.1177/20494637231159502. 37538944 PMC10395391

[nop270644-bib-0034] Shin, H. S. , J. H. Kim , and E. S. Ji . 2018. “Clinical Nurses' Resilience Skills for Surviving in a Hospital Setting: A Q‐Methodology Study.” Asian Nursing Research 12, no. 3: 175–181. 10.1016/j.anr.2018.06.003. 29964201

[nop270644-bib-0035] Smith, J. A. , R. Harré , and L. Van Langenhove . 1995. Rethinking Methods in Psychology. Sage.

[nop270644-bib-0036] Sosorburam, D. , Z.‐g. Wu , S.‐c. Zhang , et al. 2019. “Therapeutic Effects of Traditional Chinese Herbal Prescriptions for Primary Dysmenorrhea.” Chinese Herbal Medicines 11, no. 1: 10–19. 10.1016/j.chmed.2018.11.001.

[nop270644-bib-0037] Stephenson, W. 1953. The Study of Behavior; Q‐Technique and Its Methodology. University of Chicago Press.

[nop270644-bib-0038] Tsai, M.‐M. , F.‐C. Yang , S.‐M. Lee , and C.‐M. Huang . 2016. “Exploring the Experience of Dysmenorrhea and Life Adjustments of Women Undergoing Traditional Chinese Medicine Treatment.” Journal of Nursing 63, no. 4: 60–69. Original work published in Chinese. 10.6224/JN.63.4.60. 27492296

[nop270644-bib-0039] Tsonis, O. , F. Gkrozou , Z. Barmpalia , A. Makopoulou , and V. Siafaka . 2021. “Integrating Lifestyle Focused Approaches Into the Management of Primary Dysmenorrhea: Impact on Quality of Life.” International Journal of Women's Health 13: 327–336. 10.2147/IJWH.S264023. PMC7982556 33762855

[nop270644-bib-0040] Uçak, H. , and F. Süzer Özkan . 2022. “Traditional and Complementary Medicine Practices Used by Women With Premenstrual Syndrome [Premenstrual Sendrom Yaşayan Kadınların Kullandıkları Geleneksel ve Tamamlayıcı Tıp Uygulamaları].” Konuralp Medical Journal 14, no. 1: 23–29. 10.18521/ktd.778758.

[nop270644-bib-0041] Watts, S. , and P. Stenner . 2012. Doing Q Methodological Research: Theory, Method and Interpretation. Sage. 10.4135/9781446251911.

[nop270644-bib-0042] World Health Organization . 2022. WHO Statement on Menstrual Health and Rights‐50th Session of the Human Rights Council Panel Discussion on Menstrual Hygiene Management, Human Rights and Gender Equality. WHO. https://www.who.int/news/item/22‐06‐2022‐who‐statement‐on‐menstrual‐health‐and‐rights.

[nop270644-bib-0043] Wu, J. 2024. Exploring Chinese Women's Menstrual Pain Experience Through a Cultural Lens. University of Ottawa.

[nop270644-bib-0044] Xie, Y. , J. Zhao , S. Fan , et al. 2026. “Efficacy Comparison of Different External Traditional Chinese Medicine Therapies as Monotherapy or in Combination for Premenstrual Syndrome: A Systematic Review and Network Meta‐Analysis.” Frontiers in Psychiatry 17: 1720232. 10.3389/fpsyt.2026.1720232. 41717558 PMC12913161

[nop270644-bib-0045] Yang, Y. , Y. Jia , S. Fu , et al. 2025. “The Prevalence of Premenstrual Syndrome in China: A Systematic Review and Meta‐Analyses.” Frontiers in Psychiatry 16: 1640781. 10.3389/fpsyt.2025.1640781. 41019595 PMC12461857

[nop270644-bib-0046] Yöndem, Z. N. , and N. Çıtak Bilgin . 2020. “Dysmenorrhea Among Hospital Nurses and Its Effects on Work Life.” Health Care for Women International: 1–18. 10.1080/07399332.2020.1800015. 33021897

[nop270644-bib-0047] Yoshino, O. , N. Takahashi , and Y. Suzukamo . 2022. “Menstrual Symptoms, Health‐Related Quality of Life, and Work Productivity in Japanese Women With Dysmenorrhea Receiving Different Treatments: Prospective Observational Study.” Advances in Therapy 39, no. 6: 2562–2577. 10.1007/s12325-022-02118-0. 35362862 PMC9123075

[nop270644-bib-0048] Zhou, M.‐C. , B. Liu , Q.‐t. Gao , et al. 2025. “Evaluation of Health Resources and Service Utilization in Traditional Chinese Medicine Hospitals in China Based on WHO'S Comprehensive Evaluation Model.” Health Policy and Technology 14, no. 3: 100998. 10.1016/j.hlpt.2025.100998.

