# Supplementary material for: Traditional Chinese Medical Care Application Patterns Among Nurses With Menstrual Symptoms: A Q Methodology Investigation
Source: Nurs Open. 2026 Jul 15;13(7):e70644. doi: 10.1002/nop2.70644 (PMC13371093; doi:10.1002/nop2.70644)
Supplement: Supplementary file 1 — Appendix S1: Characteristic and statistically distinguishing statements for pattern 1. Appendix S2: Characteristic and statistically distinguishing statements for pattern 2. Appendix S3: Characteristic and statistically distinguishing statements for pattern 3. Appendix S4: Characteristic and statistically distinguishing statements for pattern 4. Appendix S5: Characteristic and statistically distinguishing statements for pattern 5. [file NOP2-13-e70644-s001.docx]

| **Supplementary Appendix S1.**  Characteristic and Statistically Distinguishing Statements for Factor 1 | | |
| --- | --- | --- |
| Item No. | Q statement | Factor arrays |
| 1 | I believe that experiencing menstrual pain is a regular occurrence, and I have become accustomed to it. | 2 |
| 2 | **I experience severe menstrual pain, but it only occurs during my periods.** | **4** |
| 3 | I am feeling extremely uncomfortable, but it seems that others are unable to understand the level of my distress. | 0 |
| 4 | My menstrual symptoms are only seen as severe when they are physically visible (e.g., paleness, weakness, cold sweats). | 1 |
| 5 | **I have tried several methods, such as hot drinks, compresses, and massage, to alleviate menstrual pain; however, I have only experienced mild relief.** | **3** |
| 6 | Regular exercise and nutritional supplements have limited benefits in improving menstrual health. | 0 |
| 7 | Depending solely on regular exercise or nutritional supplements to improve menstrual health is not cost-effective. | 0 |
| 8 | I was unable to come to work because of menstrual pain. | 2 |
| 9 | **I didn't notice the problem until the pain started affecting my thinking, concentration, and productivity.** | **3** |
| 10 | Severe menstrual pain is affecting my daily life and causing anxiety. | -4 |
| 11 | Menstrual pain often affects my ability to participate in social activities. | 1 |
| 12 | **I have difficulty sleeping due to menstrual pain.** | **3** |
| 13 | Due to my dysmenorrhea, some people assume that I have poor health and workability. | -1 |
| **14** | **I don't want to take menstrual leave because I am afraid of being seen as lazy.** | **2**** |
| 15 | I prefer TCM over painkillers for relief from menstrual pain. | 0 |
| 16 | I hope that TCM therapy can alleviate my menstrual pain so that it does not affect my daily life. | 2 |
| **17** | **I am hoping to find relief from menstrual pain without relying on pain medication.** | **4**** |
| **18** | **I am using TCM to improve my fertility.** | **-4**** |
| 19 | Physical conditioning using TCM is a crucial way to reduce menstrual pain. | 0 |
| **20** | **I was recommended TCM treatment by my friends and relatives for relief from menstrual discomfort.** | **-1**** |
| **21** | **Even without a prescription, I have tried TCM regimens such as Si-Wu-Tang, Zhong-Jiang-Tang, and Sheng-Hua-Tang.** | **-3**** |
| **22** | **I am trying out acupressure and dietary supplements that have been recommended on the internet for menstrual health.** | **-3**** |
| 23 | Since TCM treatment did not provide me with immediate relief, I switched to painkillers for my menstrual pain. | -1 |
| **24** | **I am concerned about the possible negative impacts of using TCM for an extended time.** | **-3**** |
| 25 | Despite knowing that not taking my medication as prescribed will reduce its effectiveness, I still do so. | -2 |
| 26 | As I did not experience the expected effectiveness, I did not take the medications seriously. | -2 |
| 27 | I only visit a doctor when I feel unwell. | 0 |
| 28 | I am worried about the side effects of medication, so I sometimes adjust the dosage or stop taking it. | -2 |
| 29 | TCM prescriptions for lifestyle and dietary adjustments are challenging, such as avoiding late nights and cold food or drinks. | 1 |
| 30 | Due to my work schedule, I cannot go to bed early or on time. | 1 |
| 31 | Regular exercise can aid in reducing menstrual discomfort, but it can be challenging to sustain. | 0 |
| 32 | I believe menstrual pain is a warning sign of health issues that should be taken seriously. | 1 |
| 33 | I have come to accept menstrual pain as a normal aspect of my body and no longer view it as a disease. | -1 |
| 34 | My religious beliefs and the companionship of my relatives and friends help me endure my menstrual pain. | -2 |
| 35 | It is unnecessary to consume nutritional supplements during menstruation. | -1 |
| 36 | I monitor my body for changes and use self-care methods to alleviate menstrual discomfort. | 0 |
| 37 | I have realized the importance of adopting a healthy lifestyle due to the pain I experience during menstruation. | -1 |
| 38 | Due to neglecting my health conditioning, I am experiencing menstrual symptoms. | -2 |
| 39 | I am experiencing symptoms of PMS, which include weight gain, breast pain, and edema. | 2 |
| 40 | I am experiencing symptoms related to my menstrual cycle, such as dizziness, cold sweats, vomiting, or nausea. | 0 |
| 41 | I am experiencing symptoms of PMS, including mood swings, anxiety, fatigue, and irritability. | 1 |
| Note. Characteristic statements of the Factor 1 (Q-sort values of +3 and +4) are shown in bold. Distinguishing statements of Factor 1 are indicated by bold type and **(p < .01). | | |

| **Supplementary Appendix S2**. Characteristic and Statistically Distinguishing Statements for Factor 2 | | |
| --- | --- | --- |
| Item No. | Q statement | Factor arrays |
| **1** | **I believe that experiencing menstrual pain is a regular occurrence, and I have become accustomed to it.** | **-4**** |
| **2** | **I experience severe menstrual pain, but it only occurs during my periods.** | **-3**** |
| 3 | I am feeling extremely uncomfortable, but it seems that others are unable to understand the level of my distress. | -2 |
| 4 | My menstrual symptoms are only seen as severe when they are physically visible (e.g., paleness, weakness, cold sweats). | 1 |
| **5** | **I have tried several methods, such as hot drinks, compresses, and massage, to alleviate menstrual pain; however, I have only experienced mild relief.** | **-1**** |
| 6 | Regular exercise and nutritional supplements have limited benefits in improving menstrual health. | -3 |
| 7 | Depending solely on regular exercise or nutritional supplements to improve menstrual health is not cost-effective. | -4 |
| **8** | **I was unable to come to work because of menstrual pain.** | **-2**** |
| **9** | **I didn't notice the problem until the pain started affecting my thinking, concentration, and productivity.** | **1**** |
| 10 | Severe menstrual pain is affecting my daily life and causing anxiety. | -2 |
| 11 | Menstrual pain often affects my ability to participate in social activities. | 1 |
| **12** | **I have difficulty sleeping due to menstrual pain.** | **3** |
| 13 | Due to my dysmenorrhea, some people assume that I have poor health and workability. | -1 |
| 14 | I don't want to take menstrual leave because I am afraid of being seen as lazy. | 0 |
| 15 | I prefer TCM over painkillers for relief from menstrual pain. | 0 |
| 16 | I hope that TCM therapy can alleviate my menstrual pain so that it does not affect my daily life. | 2 |
| 17 | I am hoping to find relief from menstrual pain without relying on pain medication. | 2 |
| 18 | I am using TCM to improve my fertility. | 0 |
| **19** | **Physical conditioning using TCM is a crucial way to reduce menstrual pain.** | **1**** |
| 20 | I was recommended TCM treatment by my friends and relatives for relief from menstrual discomfort. | 0 |
| 21 | Even without a prescription, I have tried TCM regimens such as Si-Wu-Tang, Zhong-Jiang-Tang, and Sheng-Hua-Tang. | 0 |
| 22 | I am trying out acupressure and dietary supplements that have been recommended on the internet for menstrual health. | -1 |
| 23 | Since TCM treatment did not provide me with immediate relief, I switched to painkillers for my menstrual pain. | -1 |
| 24 | I am concerned about the possible negative impacts of using TCM for an extended time. | 0 |
| 25 | Despite knowing that not taking my medication as prescribed will reduce its effectiveness, I still do so. | -2 |
| 26 | As I did not experience the expected effectiveness, I did not take the medications seriously. | -1 |
| 27 | I only visit a doctor when I feel unwell. | 0 |
| 28 | I am worried about the side effects of medication, so I sometimes adjust the dosage or stop taking it. | -2 |
| 29 | TCM prescriptions for lifestyle and dietary adjustments are challenging, such as avoiding late nights and cold food or drinks. | 1 |
| 30 | Due to my work schedule, I cannot go to bed early or on time. | 2 |
| **31** | **Regular exercise can aid in reducing menstrual discomfort, but it can be challenging to sustain.** | **2**** |
| **32** | **I believe menstrual pain is a warning sign of health issues that should be taken seriously.** | **4**** |
| 33 | I have come to accept menstrual pain as a normal aspect of my body and no longer view it as a disease. | -3 |
| 34 | My religious beliefs and the companionship of my relatives and friends help me endure my menstrual pain. | -1 |
| 35 | It is unnecessary to consume nutritional supplements during menstruation. | 0 |
| **36** | **I monitor my body for changes and use self-care methods to alleviate menstrual discomfort.** | **2**** |
| **37** | **I have realized the importance of adopting a healthy lifestyle due to the pain I experience during menstruation.** | **4**** |
| 38 | Due to neglecting my health conditioning, I am experiencing menstrual symptoms. | 0 |
| 39 | I am experiencing symptoms of PMS, which include weight gain, breast pain, and edema. | 3 |
| **40** | **I am experiencing symptoms related to my menstrual cycle, such as dizziness, cold sweats, vomiting, or nausea.** | **1**** |
| **41** | **I am experiencing symptoms of PMS, including mood swings, anxiety, fatigue, and irritability.** | **3** |
|  | | |

Note. Characteristic statements of the Factor 2 (Q-sort values of +3 and +4) are shown in bold. Distinguishing statements of Factor 2 are indicated by bold type and **(p < .01).

| **Supplementary Appendix S3.** Characteristic and Statistically Distinguishing Statements for Factor 3 | | |
| --- | --- | --- |
| Item No. | Q statement | Factor arrays |
| 1 | I believe that experiencing menstrual pain is a regular occurrence, and I have become accustomed to it. | 0 |
| 2 | I experience severe menstrual pain, but it only occurs during my periods. | 0 |
| 3 | I am feeling extremely uncomfortable, but it seems that others are unable to understand the level of my distress. | 2 |
| **4** | **My menstrual symptoms are only seen as severe when they are physically visible (e.g., paleness, weakness, cold sweats).** | **2**** |
| 5 | I have tried several methods, such as hot drinks, compresses, and massage, to alleviate menstrual pain; however, I have only experienced mild relief. | 3 |
| **6** | **Regular exercise and nutritional supplements have limited benefits in improving menstrual health.** | **2**** |
| **7** | **Depending solely on regular exercise or nutritional supplements to improve menstrual health is not cost-effective.** | **1**** |
| 8 | I was unable to come to work because of menstrual pain. | -4 |
| 9 | I didn't notice the problem until the pain started affecting my thinking, concentration, and productivity. | -1 |
| 10 | Severe menstrual pain is affecting my daily life and causing anxiety. | -2 |
| 11 | Menstrual pain often affects my ability to participate in social activities. | 0 |
| 12 | I have difficulty sleeping due to menstrual pain. | -2 |
| 13 | Due to my dysmenorrhea, some people assume that I have poor health and workability. | -2 |
| 14 | I don't want to take menstrual leave because I am afraid of being seen as lazy. | -1 |
| **15** | **I prefer TCM over painkillers for relief from menstrual pain.** | **4** |
| **16** | **I hope that TCM therapy can alleviate my menstrual pain so that it does not affect my daily life.** | **3** |
| 17 | I am hoping to find relief from menstrual pain without relying on pain medication. | 1 |
| **18** | **I am using TCM to improve my fertility.** | **4**** |
| 19 | Physical conditioning using TCM is a crucial way to reduce menstrual pain. | 3 |
| 20 | I was recommended TCM treatment by my friends and relatives for relief from menstrual discomfort. | 1 |
| 21 | Even without a prescription, I have tried TCM regimens such as Si-Wu-Tang, Zhong-Jiang-Tang, and Sheng-Hua-Tang. | 1 |
| 22 | I am trying out acupressure and dietary supplements that have been recommended on the internet for menstrual health. | 2 |
| 23 | Since TCM treatment did not provide me with immediate relief, I switched to painkillers for my menstrual pain. | 0 |
| 24 | I am concerned about the possible negative impacts of using TCM for an extended time. | 0 |
| 25 | Despite knowing that not taking my medication as prescribed will reduce its effectiveness, I still do so. | 0 |
| 26 | As I did not experience the expected effectiveness, I did not take the medications seriously. | 1 |
| 27 | I only visit a doctor when I feel unwell. | -1 |
| 28 | I am worried about the side effects of medication, so I sometimes adjust the dosage or stop taking it. | 0 |
| 29 | TCM prescriptions for lifestyle and dietary adjustments are challenging, such as avoiding late nights and cold food or drinks. | 0 |
| 30 | Due to my work schedule, I cannot go to bed early or on time. | 2 |
| 31 | Regular exercise can aid in reducing menstrual discomfort, but it can be challenging to sustain. | 1 |
| 32 | I believe menstrual pain is a warning sign of health issues that should be taken seriously. | 0 |
| 33 | I have come to accept menstrual pain as a normal aspect of my body and no longer view it as a disease. | -1 |
| 34 | My religious beliefs and the companionship of my relatives and friends help me endure my menstrual pain. | -2 |
| 35 | It is unnecessary to consume nutritional supplements during menstruation. | -2 |
| 36 | I monitor my body for changes and use self-care methods to alleviate menstrual discomfort. | -1 |
| 37 | I have realized the importance of adopting a healthy lifestyle due to the pain I experience during menstruation. | -1 |
| 38 | Due to neglecting my health conditioning, I am experiencing menstrual symptoms. | -3 |
| 39 | I am experiencing symptoms of PMS, which include weight gain, breast pain, and edema. | -3 |
| 40 | I am experiencing symptoms related to my menstrual cycle, such as dizziness, cold sweats, vomiting, or nausea. | -3 |
| **41** | **I am experiencing symptoms of PMS, including mood swings, anxiety, fatigue, and irritability.** | **-4**** |
| Note. Characteristic statements of the Factor 3 (Q-sort values of +3 and +4) are shown in bold. Distinguishing statements of Factor 3 are indicated by bold type and **(p < .01). | | |

| **Supplementary Appendix S4.** Characteristic and Statistically Distinguishing Statements for Factor 4 | | |
| --- | --- | --- |
| Item No. | Q statement | Factor arrays |
| 1 | I believe that experiencing menstrual pain is a regular occurrence, and I have become accustomed to it. | -1 |
| 2 | I experience severe menstrual pain, but it only occurs during my periods. | 2 |
| 3 | I am feeling extremely uncomfortable, but it seems that others are unable to understand the level of my distress. | -3 |
| 4 | My menstrual symptoms are only seen as severe when they are physically visible (e.g., paleness, weakness, cold sweats). | 0 |
| 5 | I have tried several methods, such as hot drinks, compresses, and massage, to alleviate menstrual pain; however, I have only experienced mild relief. | 2 |
| 6 | Regular exercise and nutritional supplements have limited benefits in improving menstrual health. | 0 |
| 7 | Depending solely on regular exercise or nutritional supplements to improve menstrual health is not cost-effective. | 0 |
| 8 | I was unable to come to work because of menstrual pain. | 2 |
| **9** | **I didn't notice the problem until the pain started affecting my thinking, concentration, and productivity.** | **4** |
| **10** | **Severe menstrual pain is affecting my daily life and causing anxiety.** | **2**** |
| **11** | **Menstrual pain often affects my ability to participate in social activities.** | **3**** |
| 12 | I have difficulty sleeping due to menstrual pain. | -1 |
| **13** | **Due to my dysmenorrhea, some people assume that I have poor health and workability.** | **2**** |
| 14 | I don't want to take menstrual leave because I am afraid of being seen as lazy. | 1 |
| 15 | I prefer TCM over painkillers for relief from menstrual pain. | 4 |
| 16 | I hope that TCM therapy can alleviate my menstrual pain so that it does not affect my daily life. | 1 |
| **17** | **I am hoping to find relief from menstrual pain without relying on pain medication.** | **-1**** |
| 18 | I am using TCM to improve my fertility. | -1 |
| **19** | **Physical conditioning using TCM is a crucial way to reduce menstrual pain.** | **3** |
| **20** | **I was recommended TCM treatment by my friends and relatives for relief from menstrual discomfort.** | **3**** |
| 21 | Even without a prescription, I have tried TCM regimens such as Si-Wu-Tang, Zhong-Jiang-Tang, and Sheng-Hua-Tang. | 1 |
| 22 | I am trying out acupressure and dietary supplements that have been recommended on the internet for menstrual health. | 0 |
| 23 | Since TCM treatment did not provide me with immediate relief, I switched to painkillers for my menstrual pain. | -1 |
| 24 | I am concerned about the possible negative impacts of using TCM for an extended time. | 0 |
| 25 | Despite knowing that not taking my medication as prescribed will reduce its effectiveness, I still do so. | -2 |
| 26 | As I did not experience the expected effectiveness, I did not take the medications seriously. | -2 |
| 27 | I only visit a doctor when I feel unwell. | 0 |
| 28 | I am worried about the side effects of medication, so I sometimes adjust the dosage or stop taking it. | 0 |
| **29** | **TCM prescriptions for lifestyle and dietary adjustments are challenging, such as avoiding late nights and cold food or drinks.** | **-2**** |
| **30** | **Due to my work schedule, I cannot go to bed early or on time.** | **-3**** |
| 31 | Regular exercise can aid in reducing menstrual discomfort, but it can be challenging to sustain. | 0 |
| 32 | I believe menstrual pain is a warning sign of health issues that should be taken seriously. | -1 |
| 33 | I have come to accept menstrual pain as a normal aspect of my body and no longer view it as a disease. | 1 |
| 34 | My religious beliefs and the companionship of my relatives and friends help me endure my menstrual pain. | -3 |
| 35 | It is unnecessary to consume nutritional supplements during menstruation. | -4 |
| 36 | I monitor my body for changes and use self-care methods to alleviate menstrual discomfort. | -2 |
| 37 | I have realized the importance of adopting a healthy lifestyle due to the pain I experience during menstruation. | 1 |
| 38 | Due to neglecting my health conditioning, I am experiencing menstrual symptoms. | -2 |
| 39 | I am experiencing symptoms of PMS, which include weight gain, breast pain, and edema. | 0 |
| 40 | I am experiencing symptoms related to my menstrual cycle, such as dizziness, cold sweats, vomiting, or nausea. | -4 |
| 41 | I am experiencing symptoms of PMS, including mood swings, anxiety, fatigue, and irritability. | 1 |
| Note. Characteristic statements of the Factor 4 (Q-sort values of +3 and +4) are shown in bold. Distinguishing statements of Factor 4 are indicated by bold type and **(p < .01). | | |

| **Supplementary Appendix S5.** Characteristic and Statistically Distinguishing Statements for Factor 5 | | |
| --- | --- | --- |
| Item No. | Q statement | Factor arrays |
| 1 | I believe that experiencing menstrual pain is a regular occurrence, and I have become accustomed to it. | 2 |
| **2** | **I experience severe menstrual pain, but it only occurs during my periods.** | **3** |
| 3 | I am feeling extremely uncomfortable, but it seems that others are unable to understand the level of my distress. | -3 |
| **4** | **My menstrual symptoms are only seen as severe when they are physically visible (e.g., paleness, weakness, cold sweats).** | **-2**** |
| **5** | **I have tried several methods, such as hot drinks, compresses, and massage, to alleviate menstrual pain; however, I have only experienced mild relief.** | **4** |
| 6 | Regular exercise and nutritional supplements have limited benefits in improving menstrual health. | -3 |
| 7 | Depending solely on regular exercise or nutritional supplements to improve menstrual health is not cost-effective. | -3 |
| 8 | I was unable to come to work because of menstrual pain. | -4 |
| 9 | I didn't notice the problem until the pain started affecting my thinking, concentration, and productivity. | 0 |
| 10 | Severe menstrual pain is affecting my daily life and causing anxiety. | -2 |
| 11 | Menstrual pain often affects my ability to participate in social activities. | 0 |
| 12 | I have difficulty sleeping due to menstrual pain. | -2 |
| 13 | Due to my dysmenorrhea, some people assume that I have poor health and workability. | -2 |
| 14 | I don't want to take menstrual leave because I am afraid of being seen as lazy. | 0 |
| **15** | **I prefer TCM over painkillers for relief from menstrual pain.** | **2**** |
| 16 | I hope that TCM therapy can alleviate my menstrual pain so that it does not affect my daily life. | 2 |
| **17** | **I am hoping to find relief from menstrual pain without relying on pain medication.** | **3** |
| **18** | **I am using TCM to improve my fertility.** | **-4**** |
| 19 | Physical conditioning using TCM is a crucial way to reduce menstrual pain. | 2 |
| 20 | I was recommended TCM treatment by my friends and relatives for relief from menstrual discomfort. | 0 |
| 21 | Even without a prescription, I have tried TCM regimens such as Si-Wu-Tang, Zhong-Jiang-Tang, and Sheng-Hua-Tang. | 2 |
| 22 | I am trying out acupressure and dietary supplements that have been recommended on the internet for menstrual health. | -1 |
| 23 | Since TCM treatment did not provide me with immediate relief, I switched to painkillers for my menstrual pain. | -1 |
| 24 | I am concerned about the possible negative impacts of using TCM for an extended time. | -1 |
| 25 | Despite knowing that not taking my medication as prescribed will reduce its effectiveness, I still do so. | 1 |
| 26 | As I did not experience the expected effectiveness, I did not take the medications seriously. | 0 |
| **27** | **I only visit a doctor when I feel unwell.** | **1**** |
| 28 | I am worried about the side effects of medication, so I sometimes adjust the dosage or stop taking it. | -1 |
| 29 | TCM prescriptions for lifestyle and dietary adjustments are challenging, such as avoiding late nights and cold food or drinks. | -1 |
| 30 | Due to my work schedule, I cannot go to bed early or on time. | 0 |
| 31 | Regular exercise can aid in reducing menstrual discomfort, but it can be challenging to sustain. | 1 |
| 32 | I believe menstrual pain is a warning sign of health issues that should be taken seriously. | 1 |
| 33 | I have come to accept menstrual pain as a normal aspect of my body and no longer view it as a disease. | 1 |
| 34 | My religious beliefs and the companionship of my relatives and friends help me endure my menstrual pain. | -1 |
| 35 | It is unnecessary to consume nutritional supplements during menstruation. | 0 |
| 36 | I monitor my body for changes and use self-care methods to alleviate menstrual discomfort. | 0 |
| 37 | I have realized the importance of adopting a healthy lifestyle due to the pain I experience during menstruation. | 1 |
| **38** | **Due to neglecting my health conditioning, I am experiencing menstrual symptoms.** | **0**** |
| 39 | I am experiencing symptoms of PMS, which include weight gain, breast pain, and edema. | 3 |
| 40 | I am experiencing symptoms related to my menstrual cycle, such as dizziness, cold sweats, vomiting, or nausea. | -2 |
| **41** | **I am experiencing symptoms of PMS, including mood swings, anxiety, fatigue, and irritability.** | **4** |
| Note. Characteristic statements of the Factor 5 (Q-sort values of +3 and +4) are shown in bold. Distinguishing statements of Factor 5 are indicated by bold type and **(p < .01). | | |

|  |  |  |
| --- | --- | --- |
